# Competition of multiplatform firms: Implications for the Internet of Things

**DOI:** 10.1371/journal.pone.0300522

**Published:** 2024-05-14

**Authors:** Frank MacCrory, Evangelos Katsamakas

**Affiliations:** 1 Merage School of Business, University of California Irvine, Irvine, California, United States of America; 2 Gabelli School of Business, Fordham University, New York, New York, United States of America; Beijing University of Technology, CHINA

## Abstract

The Internet of Things (IoT) technology trend is transforming business and society. This creates a need to understand strategic behavior in the consumer IoT, where firms tend to offer multiple platform devices, and new generations of devices are introduced frequently. We propose a novel analytical model that formalizes the concept of a *multiplatform firm* that offers a *system of platforms*, such as a smartphone, and a new platform device, such as a smartwatch, and orchestrates a *multiplatform ecosystem*. The analysis shows how a platform design decision, like offering a new standalone device, affects consumer choices and market outcomes. We identify two classes of new devices that matter, and show when a new platform device may disrupt the smartphone market. Moreover, we characterize conditions under which it is profitable for a vendor to make its new platform device look and feel more like its smartphone. Overall, we provide insights into how multiplatform firms differ from platform firms. We identify future research opportunities on the economics of consumer IoT and multiplatform ecosystems.

## 1. Introduction

The Internet of Things (IoT) concept refers to everyday objects that feature processing power, software, sensors, and actuators while interconnecting with the rest of the Internet [1]. IoT is one of the most significant contemporary technology and business trends that promise to transform business [2, 3]. IoT promises the integration of physical and digital worlds and the creation of new value. It could make supply chains more efficient to transform retail, public auditing, and several other industries. Smart IoT devices, including smartwatches (*e*.*g*., Apple Watch) and smart home appliances (*e*.*g*., Amazon Echo) create new value for consumers.

Smart, connected products are transforming companies and competition [4, 5]. New IoT products and markets [6, 7] and new IoT business models [8, 9] are expected to have a significant business and economic impact. Firms may offer several devices, and each device could be a platform that enables device users to interact with applications (apps) developed by outside developers. Moreover, the consumer IoT landscape rapidly evolves as vendors offer new devices and capabilities. For example, Apple introduced Apple Watch in 2015 and releases a new series yearly—the latest Series 9 was released in September 2023 (https://www.apple.com/watch/). However, there exists very little economic modeling research on IoT [10], and our work aims to fill this research gap.

We propose an analytical model of competition motivated by competition in the consumer IoT. We consider two competing firms, each offering two platform devices, *i*.*e*., first a smartphone and then adding another smart platform device. Devices of the same type are horizontally differentiated. A firm that offers multiple platform devices is a *multiplatform firm*. It offers a *system of platforms* and orchestrates a *multiplatform ecosystem* that consists of all the users and app developers for all the platform devices offered by the firm. Our article formalizes the multiplatform ecosystem concept and proposes a novel analytical model of competition of multiplatform ecosystems. Our article characterizes strategic behavior in this setting and shows how firms’ multiplatform design choices affect consumer choices and market outcomes. The main questions we answer are: How do *multiplatform firms* differ from platform firms? How do firms’ new platform device choices affect competition? When does the new platform device start disrupting the smartphone market, such that consumers cease adopting the smartphone?

Because we want to capture how firm decisions affect outcomes in the consumer IoT landscape, we identify and formalize distinct competition cases. In Case 1, each firm offers only one platform device, such as an Android smartphone. In Case 2, firms coordinate two distinct platform devices each, though the second device works only with a matching first device from the same vendor, such as an iPhone and Apple Watch. In Case 3, each firm coordinates two distinct platform devices that can operate independently of one another, such as a Fire tablet and an Echo smart speaker (the Echo can be set up with any of several connected devices, including Android and iPhone, then operates independently).

Each of these devices serves as the consumer-side IoT “thing” that presents options (in this case, apps) provided by a developer side, creating a two-sided market that benefits from third-party effort on both sides. Developers’ efforts directly broaden the selection of available apps and the marketing of their apps, while consumer efforts through reviews enhance matching and word-of-mouth draws additional consumers to the two-sided market. Meanwhile, the firm owning the platform facilitates search, matching, transactions, and dispute resolution.

We show that the “second” platform device in each firm’s ecosystem of platforms has a differing effect that depends on the relative strength of consumer tastes for those “second” devices relative to the smartphone. The “second” platform device plays an important role in firm strategies and affects competitive market outcomes. Most importantly, we show that if a “second” platform device is standalone, the device may start disrupting the smartphone market—some consumers will adopt the “second” device and cease using a smartphone.

## 2. Background

Overall, previous analytical modeling literature examines multiple strategic considerations of platform firms and platform competition [11, 12]. A platform firm provides an infrastructure that facilitates the interaction between two or more groups of parties who would otherwise have significant (and perhaps insurmountable) friction transacting on their own. Examples of platform markets are widely varied and include real estate matching homebuyers and home sellers, dating/matchmaking services, game consoles matching game developers and gamers, credit card firms matching merchants and customers, and stores that allow suppliers to set up kiosks to reach customers directly (printer companies in electronics stores, cosmetics companies in departments stores, *etc*.). A platform firm needs to consider the value created in facilitated transactions (also known as cross-side network effects) and set a price structure and price level that maximizes the platform profit [13–19]. Other important issues concerning platform firms include openness [20–25], seller competition on a platform [26], multihoming [27], platform envelopment [28], reseller models [29], new products and sellers [30], platforms hosting rival firms [31], investments in platform integration [32], and changing production costs over time [33]. However, there is a lack of analytical work on multiplatform firms.

Motivated by the consumer IoT context, this article contributes to the platform economics literature because it analyzes how the strategies of *multiplatform firms* differ from those of traditional platform firms. A *multiplatform firm* coordinates a *multiplatform ecosystem*. Strategic behavior in the context of multiplatform ecosystems is a novel research phenomenon. A conventional platform firm must consider the interaction between the platform sides (cross-side network effects) and competition from other platform firms. A multiplatform firm, however, must consider the interaction between the sides of each platform it offers, the interaction between the platforms in their *system of platforms*, and competition from other platform or multiplatform firms.

Our analytical model is presented next, followed by the results, discussion, and conclusions.

## 3. Model

We consider two firms, A and B, competing in the consumer IoT by selling platform devices. Developers build apps for the platform devices, and Consumers adopt devices to benefit from using the devices and their apps. Developers are allowed to multi-home, for instance, develop apps for Phone A and Phone B. Consumers purchase at most one of each product type (*e*.*g*., they single-home).

### 3.1 Competition Cases for Firms A and B

In the baseline Case 1, each firm sells only a smartphone that we will label as Phones. Smartphones are platform devices: they connect app Developers with Consumers to create positive cross-side network effects. Developers benefit when more Consumers use the smartphone (larger potential market for sales), and Consumers benefit when more Developers develop apps for the smartphone (wider variety of apps allowing better fit to Consumer tastes).

Firms A and B may decide to introduce new platform devices that we will label as Watches. The new platform devices connect app Developers with Consumers to create positive cross-side network effects: as with Phones, Developers benefit when more Consumers use the new platform device, and Consumers benefit when more Developers develop apps for the platform device. If Watches are tethered to the Phone then we have Case 2, but if the new platform devices can function as standalone devices, then we have Case 3.

In Case 2, the new platform device is tethered to the Phone from the same firm. This means that a consumer can use a Watch only if they have the matching Phone. Formally, a Watch has zero utility without a matching Phone (Leontief complementarity). In Case 2, mixed-vendor systems are not feasible because the Consumer would get no utility from the Watch.

In Case 3, firms offer a new platform device that can function as a standalone device. This permits consumers to mix and match vendors if desired, so mixed-vendor systems are feasible in the market (*e*.*g*., purchase Phone A and Watch B).

**Fig 1:**
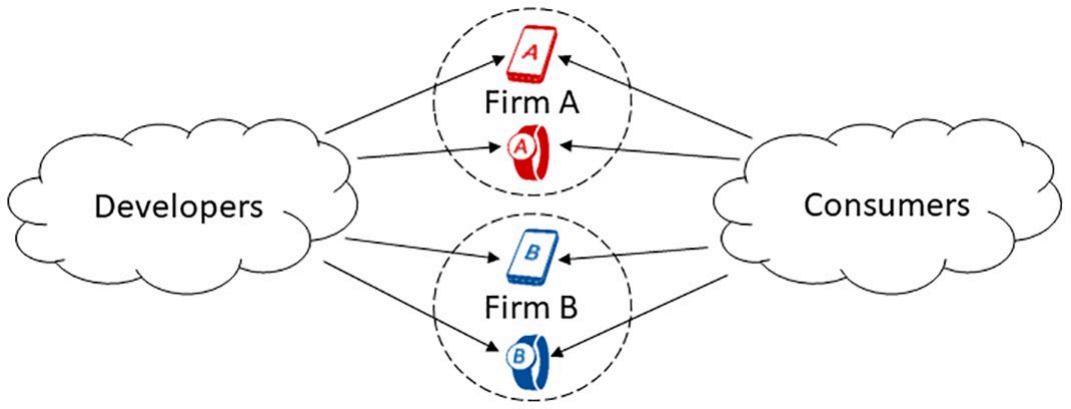
Illustration of developer and consumer choices between competing multiplatform Firms.

While in Case 1 firms A and B are platform firms, in Cases 2 and 3, firm A is a *multiplatform firm* and firm B is a competing *multiplatform firm*. Each firm offers a *system of platforms*, and each firm orchestrates a *multiplatform ecosystem*. Our model considers how those *multiplatform firms* compete under different cases. Fig 1 illustrates these concepts.

In all Cases, Firms A and B maximize their profits, as shown in Eq (1), from the platform device(s) they offer in a particular Case. We analyze the equilibrium outcome of each Case rather than the market-share-building phase, often characterized by incremental entry into markets and/or unsustainable heavy discounting. For each platform device that Firms offer, they set a price for Consumers to purchase the device and an access price for Developers.


$$\Pi_{k}=P_{Pk}∙Q_{Pk}+p_{Pk}∙q_{Pk}+P_{Wk}∙Q_{Wk}+p_{Wk}∙q_{Wk}$$ (1)

Capitalized variables refer to Consumer price and quantity, while lowercase variables refer to Developer price and quantity. See Table 1 for a summary of all notation.

**Table 1:** Summary of key notation.

{*i*,|*j*}	Consumer adoption pattern with Watch from firm *i∈*{A,B,∅} and Phone from firm *j∈*{A,B,∅}, where ∅ stands for no purchase (outside option)
*N*_*P*_, *N*_*W*_	Cross-side network effects enjoyed by Consumers for Phone, and Watch
*n*_*P*_, *n*_*W*_	Cross-side network effects enjoyed by Developers for Phone, and Watch
*P*_*Pi*_,*P*_*Wi*_	Prices paid by Consumers to acquire the device from firm *i*
*p*_*Pi*_,*p*_*Wi*_	Prices paid by Developers to access the device from firm *i*
*Q*_*Pi*_, *Q*_*Wi*_	Quantity (market share) of Consumers for the device from firm *i*
*q*_*Pi*_, *q*_*Wi*_	Quantity (market share) of Developers for the device from firm *i*
*T*_*P*_, *T*_*W*_	Transport/misfit cost for Consumers for Phone, and Watch
*t*_*P*_, *t*_*W*_	Transport/misfit cost for Developers for Phone, and Watch
*U*_*i*,*j*_	Total utility to Consumer for acquiring Watch *i* and Phone *j*
*u*_*P*_, *u*_*W*_	Intrinsic consumer value of acquiring a Phone, and Watch, respectively
*u*_*Pi*_, *u*_*Wi*_	Utility for Consumer from the device from firm *i*
*V*_*i*,*j*_	Total value to Developer for accessing Watch *i* and Phone *j*
*v*_*P*_, *v*_*W*_	Intrinsic value of developing an app for Phone, and Watch
*v*_*Pi*_, *v*_*Wi*_	Value for Developer from the device from firm *i*
*x*	Index of Watch taste with range [0,1]
*y*	Index of Phone taste with range [0,1]

### 3.2 Consumers

We now focus on Consumer preferences for Phones and new platform devices (Watches). Consumers have unit demand for at most one Phone and at most one Watch. This means that they single-home separately for each type of device (e.g., a Consumer that buys a Phone from firm A will not buy a Phone from firm B, and a Consumer that buys a Watch from firm B will not buy a Watch from A).

**Fig 2:**
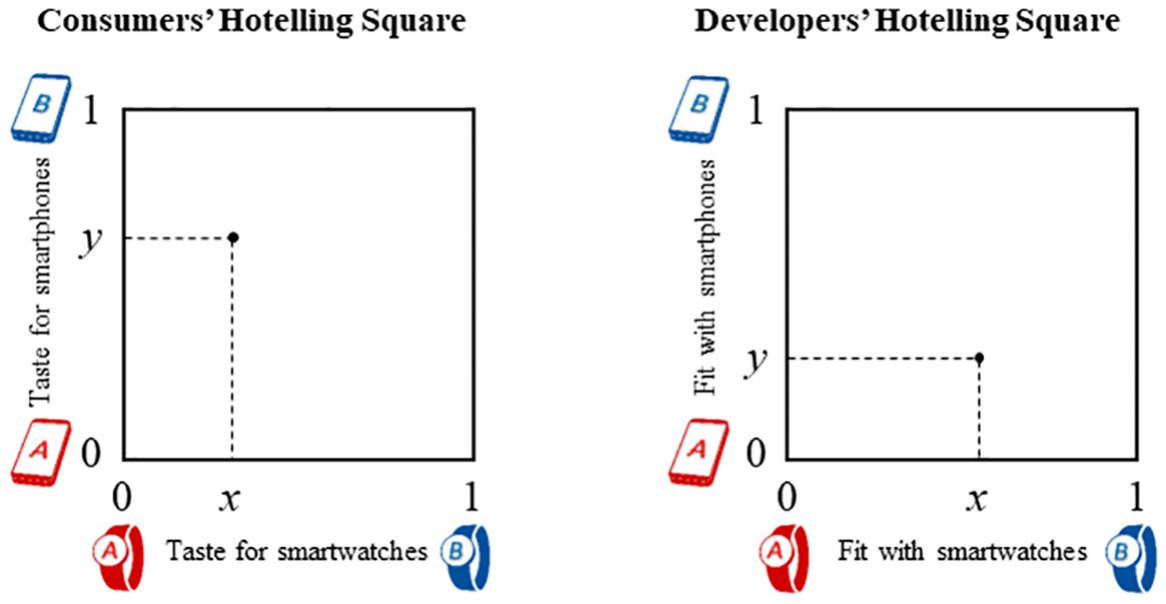
(a) Consumers’ Hotelling square of preferences for using Phones and Watches, and (b) Developers’ Hotelling square of preferences for building apps for Phones and Watches.

Consumers are represented in a *Consumers’ Hotelling square*: They have horizontal preferences for devices that we model as uniformly distributed locations in a Hotelling square (Fig 2a), with the *x*-axis related to Watches and the *y*-axis related to Phones. Devices from Firm A are normalized to location 0, and devices from Firm B are normalized to location 1. Distance from a device represents a misfit cost that might arise from the Consumer’s tastes for specific features, form factors, and aesthetics. The example Consumer in Fig 2a has a distinct preference for Watch A over Watch B but prefers Phone B only slightly over Phone A.

We index a Consumer’s adoption {*i*, *j*} by the Watch *i* and Phone *j* purchased from Firm A, Firm B, or ∅ (no purchase). Then, the utility function of a Consumer located at (*x*, *y*) in the Consumers’ Hotelling square adopting {*i*, *j*} is shown in Eq (2). For our base model, utility is additively separable in utility from the Watch *u*_*Wi*_, utility from the Phone *u*_*Pj*_, and prices *P*_*Wi*_ and *P*_*Pj*_. We consider interrelated utility in the Model Extension Section after the main model.


$$U_{i,i}\left(x,y\right)=u_{Wi}\left(x\right)+u_{Pj}\left(y\right)-P_{Wi}-P_{Pj}$$ (2)

The component utilities from the Watch and Phone derive from an intrinsic utility of the device itself as if it was the Consumer’s ideal device (*u*_*W*_ and *u*_*P*_), cross-side networks effects (*N*_*W*_ and *N*_*P*_) scaled by the quantity of Developers associated with each device (*q*_*Wi*_ and *q*_*Pj*_), and disutility (*T*_*W*_ and *T*_*P*_) scaled by the difference between a Consumer’s ideal device (*x* and *y*) and the actual device.


$$\begin{array}{ll} u_{WA}\left(x\right)=u_{W}+N_{W}∙q_{WA}-x∙T_{W} & u_{PA}\left(y\right)=u_{W}+N_{W}∙q_{WA}-y∙T_{P}\\ u_{WB}\left(x\right)=u_{W}+N_{W}∙q_{WB}-\left(1-x\right)∙T_{W} & u_{PB}\left(y\right)=u_{W}+N_{W}∙q_{WB}-\left(1-y\right)∙T_{P} \end{array}$$

The utility of the outside option ∅ (non-purchase) is normalized to zero, and it will be chosen by a Customer in lieu of a Phone or Watch that would yield negative utility.

### 3.3 App developers

Developers develop apps to maximize their profit from apps. Developers are able to multi-home, so they can develop apps for any combination of platform devices available in the market in a particular competition case. For example, a Developer may develop apps for Phone A, and Phone B, as well as Watch B.

Developers are heterogeneous with respect to the cost of developing applications, and they are uniformly distributed across a *Developers’ Hotelling square* (Fig 2), with the *x*-axis related to fit with specific Watches and the *y*-axis related to fit with specific Phones. The distance along a dimension between the Developer and the device creates a misfit cost that might arise from the availability of sensors, the device’s form factor, the vendor’s policies on content, and the platform’s security posture. The example Developer depicted in Fig 2b has app ideas that have almost identical levels of misfit with each Watch, but can develop apps for Phone A relatively easily.

The value function of a Developer located at (*x*, *y*) in Developers’ Hotelling square is shown in Eq (3). Value is additively separable in each device for which the Developer chooses to create apps, and each of these is, in turn, additively separable in economic value to the Developer (*v*) and prices paid to the platform (*p*). While the Developers pay fixed prices in our model, since there is no uncertainty, a fixed price is mathematically equivalent to a known commission, such as those charged by Google Play and the Apple App Store.


$$V_{i,j}\left(x,y\right)=\left(\sum_{i=\left\{A,B\right\}}\;\text{max}\left[v_{Wi}\left(x\right)-p_{Wi},0\right]\right)+\left(\underset{i=\left\{A,B\right\}}{\sum }\;\text{max}\left[v_{Pj}\left(x\right)-p_{Pj},0\right]\right)$$ (3)

The component value for each device derives from an intrinsic value reprsented by *v*_*W*_ and *v*_*P*_ of developing the app itself (*e*.*g*., knowledge and digital assets less cost of effort) for an ideal device, cross-side network effects (marginal revenue from sales) represented by *n*_*W*_ and *n*_*P*_, scaled by the quantity of Consumers on that device represented by *Q*_*Wi*_ and *Q*_*Pj*_, and disutility (*t*_*W*_ and *t*_*P*_) scaled by the difference between a Developer’s ideal device (*x* and *y*) and the actual device.


$$\begin{array}{ll} v_{WA}\left(x\right)=v_{W}+n_{W}∙Q_{WA}-x∙t_{W} & v_{PA}\left(y\right)=v_{P}+n_{P}∙Q_{PA}-y∙t_{P}\\ v_{WB}\left(x\right)=v_{W}+n_{W}∙Q_{WB}-\left(1-x\right)∙t_{W} & v_{PB}\left(y\right)=v_{P}+n_{P}∙Q_{PB}-\left(1-y\right)∙t_{P} \end{array}$$

Market shares such as *Q*_*WA*_ are endogenously determined and range over [0, 1]. Cross-side network effects are proportional to market share on the other side of the network.

### 3.4 Defining classes of new platform devices

We define two classes of new platform devices, depending on the strength of preferences for those new devices.

**Definition 1 (Strong taste devices):** A Strong taste device is a platform device for which tastes are stronger than Phone tastes such that misfit costs have the relationship *T*_*W*_ > *T*_*P*_ and *t*_*W*_ ≥ *t*_*P*_.■

**Definition 2 (Weak taste devices):** A Weak taste device is a platform device for which tastes are weaker than Phone tastes such that *T*_*W*_ < *T*_*P*_ and *t*_*w*_ ≤ *t*_*P*_.■

Real devices may fall into one of those classes for a variety of design reasons. For instance, tight engineering constraints of the Watch form factor lead to drastic trade-offs among desired features, so these devices are likely to be Strong taste devices. For example, watch-sized cellular radios existed when the first Watches were introduced, but battery technology was not yet advanced enough to power a cellular radio for a reasonable length of time, so firms prioritized other features.

Less-mobile devices (Alexa smart speakers, HomeKit home hubs, ADT smart security systems, *etc*.) are likely to be Weak taste devices. For example, Speakers (which are far more sophisticated smart-home coordinators than the name “Speaker” suggests) do not have serious size or power constraints. They are controlled primarily through voice commands, so horizontal differentiation of Speakers should be weaker than Phones rather than stronger.

Other devices may be more difficult to categorize. A device with a primary interface through a virtual-reality headset would face tight technical constraints like Watches, but employ very similar user interfaces that tend to depress differentiation. For devices such as these, the effective strength of tastes would be an empirical question.

## 4. Analysis and results

We present the results starting with the analysis of the baseline Case 1 (Phones only), then Case 2, and lastly Case 3. Each Case is analyzed as a static game with the steps listed in Table 2, and we solve for the Nash equilibrium that the market would eventually reach over time.

**Table 2:** Game steps for each Case (1–3).

1	Firms A and B noncooperatively set Consumer prices and Developer prices for the devices they offer.
2	Developers decide for which devices they will build and sell apps.
4	Consumers decide which devices to purchase.
5	Consumer utility, Developer value, and Firm profit determined simultaneously.

### 4.1 Baseline Case 1 (Firms A and B sell Phones only)

The only platform devices available are Phones. Therefore, the Consumers can purchase a Phone from firm A, a Phone from firm B, or decline to purchase any Phone.

We assume an interior solution for Developer market shares because this implies that some Developers sell on one platform but not the other, representing the real market for Phone app Developers. Working backward to minimal conditions that would lead to an interior solution, we place a mild constraint on transport/taste costs, assuming that Developer misfit costs are at least

tP>vp2+nP4>0 (A1)

We derive a lemma that provides the foundation for some of our later results.

**Lemma 1:** The condition for full market coverage of Phone Consumers in the presence of network effects is TP≤2uP+NPtP∙vP+nP2, which is increasing in network effects or intrinsic utility for either side of the market. Firms charge Consumers *T*_*P*_ and Developers vP2+nP4 when an interior solution obtains, serving a fraction vP2+nP4tP of Developers. Each firm’s profit is TP2 from Consumers plus vP2+nP42tP from Developers.■

All proofs are in the S1 Appendix.

**Fig 3:**
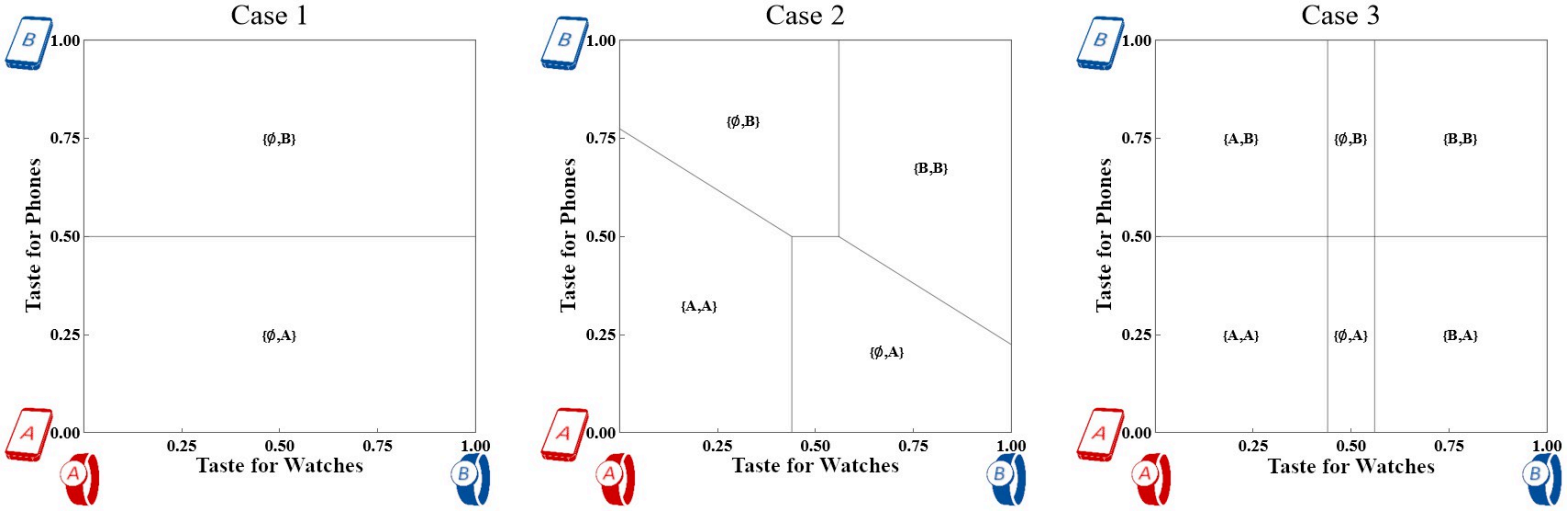
Consumer market shares across three Strong device cases with *T*_*W*_ = ^5^/_16_ and *T*_*P*_ = ^1^/_4_. The horizontal axis measures taste for Watches, while the vertical axis measures taste for Phones. Developer market shares are much simpler because Firms treat multihoming developers as if the other Firm does not exist.

The intuition behind this result is that with symmetric firms, each competes away network effects on the Consumer side and charges a price equal to the transport cost *T*_*P*_. The market outcome is shown in Fig 3. The free utility from cross-side network effects makes full coverage on the Consumer side likely. Firms attract Developers independent of their activity with the competing firm, so the prices offered to Developers are those of local monopolists. This result is observed empirically [14, 16], and appears in other competition models [34, 35].

For the figures illustrating market outcomes, we choose model parameters that ensure that all purchase combinations of interest occur within the Hotelling square. These parameters are: uP=uW=vP=vW=110;tP=tW=14;NP=NW=nP=nW=12. In the rest of the analysis, we shall assume unless otherwise noted that the condition in Lemma 1 holds, so that Consumer misfit costs are

0<TP≤2uP+NPtP+vP+nP2 (A2)

### 4.2 Case 2: Watch works only with matching phone

Firms A and B introduce a new platform device (Watch) that works only with a matching Phone. That is, Consumers may purchase {∅,A}, {A,A}, {∅,B}, {B,B}, or {∅,∅}.

We assume that the new platform device is a Strong taste device that satisfies Definition 1 above (we relax this assumption in the New Platform Devices Are Weak Taste Devices subsection).

We also assume that firms will keep the existing Phone prices that guarantee a fully covered Consumer market for the older product. Empirically, we observe that baseline smartphone prices have stayed fairly static as shown in the second figure of [36].

We will also extend the previous assumption (A1) to watch Developers, so that the Developer misfit costs are at least

tW>vP2+nP4>0 (A3)

We now introduce a definition to separate two distinct market outcomes for Case 2.

**Definition 3:** Adoption of the watch is Wide if the indifference line between systems {A,A} and {∅,A} occurs at *x* ≥ ^1^/_2_, and conversely, the indifference line between systems {B,B} and {∅,B} occurs at *x* ≤ ^1^/_2_. This requires NW≥4tW∙32TW-uWnW+2vW. Otherwise, the adoption of the watch is Narrow with NW<4tW∙32TW-uWnW+2vW.■

We derive the outcome of this market in Proposition 1.

**Proposition 1 (Market outcome in Case 2):** In Case 2, Firms charge Consumers *T*_*W*_ for Watches and Developers vW2+nW4 when an interior solution obtains, serving a fraction vW2+nW4tW of Developers. Each Firm’s profit is 40TP-20TW+920TW-91600TP from Consumers if adoption is Narrow per Definition 3 or 40TP9-20TW+TW153-260TW-8911600TP if adoption is Wide. Either profit is deterministically *T*_*W*_/2 or lower. At either level of adoptions, each Firm’s profits from Developers is vW2+nW42tW. A positive fraction Consumers purchase a phone but no Watch if Watch cross-side network effects are less than NW<4tW∙2TW-uWnW+2vW.■

If misfit costs are very low, all Consumers purchase the single-vendor systems. Otherwise, some Consumers purchase a Phone only. Although the market shares have changed shape, indicating platform switching, each Firm still sells Phones to 50% of the Consumers and sells Watches to some fraction of its Consumers. We illustrate the impact in the middle panel of Fig 3. Watches are unambiguously revenue-enhancing for both Firms.

**Fig 4:**
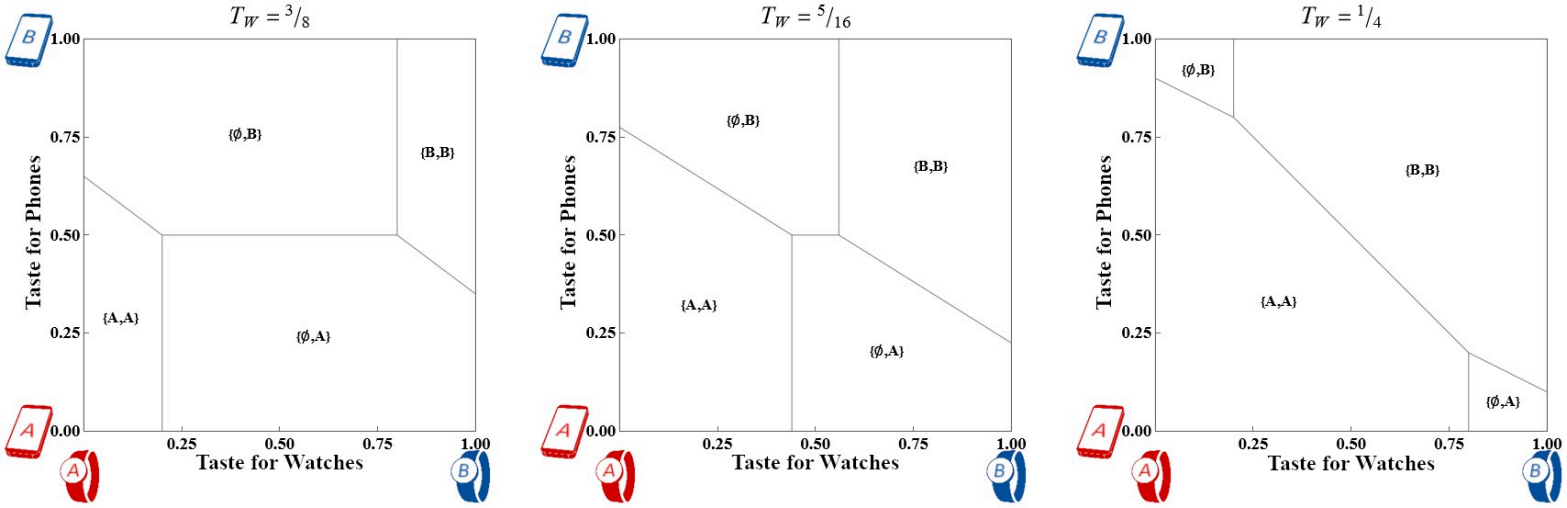
Consumer market shares in Case 2 across three levels of *T*_*W*_ with *T*_*P*_ = ^1^/_4_. The horizontal axis measures taste for Watches, while the vertical axis measures taste for Phones. The first and second panels show Narrow adoption according to Definition 3; the third panel shows Wide adoption.

The numerical example used in Fig 3 represents Narrow adoption. Fig 4 shows Case 2 at three different levels of Watch adoption including both Narrow and Wide.

We do not need to analyze in detail the outcome if only one firm introduces a Watch (or the second Watch’s introduction is significantly delayed); the firm with a Watch will steal some of the rival’s Consumer market share.

### 4.3 Case 3: Stand-alone Watches

Firms A and B introduce Watches as standalone platform devices. An example would be enhancing the Watch with independent GPS and phone-call ability (*e*.*g*., Apple Watch Series 3 or later but without requiring an iPhone for setup).

Consumers have additional options compared to Case 2: mixed-vendor system ({A,B}, {B,A}), or adopt only a new platform device ({A,∅}, {B,∅}) if this maximizes their total utility.

**Fig 5:**
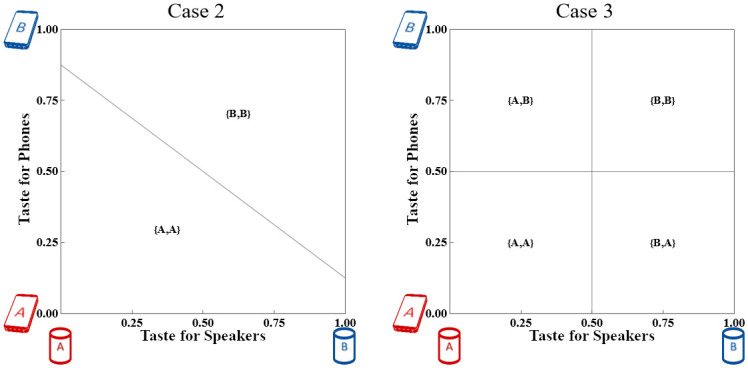
Consumer market shares of Weak taste devices with *T*_*S*_ = ^3^/_16_ and *T*_*P*_ = ^1^/_4_. The horizontal axis measures taste for Speakers while the vertical axis measures taste for Phones.

By removing the constraint to choose a particular Phone to use a preferred Watch, Case 3 induces at least as many consumers as Case 2 to purchase a Watch that meets their tastes. The right panel in Fig 5 shows the market outcome.

We now derive the conditions under which some consumers prefer a mixed-vendor system.

**Lemma 2:** Mixed-vendor systems will have a positive market share if Consumer cross-side network effects are at least NW>4tW∙TW-uwnW+2vW.■

The intuition behind this result is that this range guarantees the indifference line between {B,A} and {∅,A} is located at *x* < 1. (The other indifference line important for mixed-vendor systems is between bundles {A,A} and {B,A}. This line is always located at *x* = ^1^/_2_ and therefore always located at *x* < 1.) Note that both Narrow and Wide adoption of watches is possible under this condition. We will assume that Lemma 2 holds, and conservatively assume that network effects are not *so* strong as to cause every Phone Consumer to prefer to buy a matching Watch (see the final portion of Proposition 1’s proof in the S1 Appendix), so that Consumer cross-side network effects are in the range

4tW∙TW-uwnW+2vW<NW<4tW∙2TW-uwnW+2vW (A4)

Under those conditions, we can state the following Proposition about the market outcome.

**Proposition 2 (Market outcome in Case 3):** In Case 3, each of the four systems captures an equal market share of Consumers. Under Wide adoption of Watches per Definition 3, each captures one-quarter with indifference lines at *x* = ^1^/_2_ and *y* = ^1^/_2_. Under Narrow adoption of Watches, there exists a group of Consumers centered on *x* = ^1^/_2_ that purchase a Phone but no Watch. While technically feasible, no Consumers purchase a Watch but no Phone.■

Under Narrow adoption in Case 2, adding standalone Watches causes no Consumers to switch Watches, but some Consumers do purchase Watches who had not done so when they were only available in single-vendor systems. Some of the Consumers who purchased Case 2 systems switch to their preferred Phones, as shown in the right panel of Fig 5.

The outcome is similar under Wide adoption in Case 2, except that Case 3 sees some Consumers switching to a preferred Watch. Wide adoption in Case 2 is sufficient to guarantee Watches enjoy full Consumer market coverage in Case 3.

As with Case 2, we do not need to analyze in detail a situation in which only one Watch is independent, or an independent Watch facing no rival Watch at all.

### 4.4 New platform devices are weak taste devices

What if the new platform devices are Weak taste devices? Then assumption (A2) and Definition 2 combine to guarantee that adoption will always be Wide, so there is no need to consider cases of Narrow adoption.

The intuition is that since optimal prices can cover the Consumer market with Phone-strength tastes, optimal prices will also cover the Consumer market for the Weak taste device once the market reaches equilibrium. See Fig 5, which has the same parameter values as in Fig 5 except with *T*_*S*_ = ^3^/_16_ in place of *T*_*W*_.

### 4.5 New platform device disrupts the phone market

Under what conditions would a new platform device that is initially a complement to the Phone start disrupting the Phone market, in which case some Consumers use only the new platform device and cease adopting a Phone? We characterize the conditions under which this can occur in Proposition 3.

**Proposition 3 (New platform device disrupts the Phone):** A positive market share of Consumers who purchase the new platform device and do not purchase the Phone obtains in Case 3 if the misfit cost *T*_*P*_ is high enough that assumption (A2) is violated without losing all sales of either device.■

Note that a key condition for Proposition 3 is a high enough misfit cost for Phones. This could result either from exogenous technological constraints, or because *Firms focus on core customers’ tastes* [37] and invest to increase the transport/misfit cost (or more precisely, widen the feature space) for Phones beyond the limit in assumption (A2). Notably, the conditions for Proposition 3 can obtain whether the new device exhibits Strong or Weak tastes.

**Fig 6:**
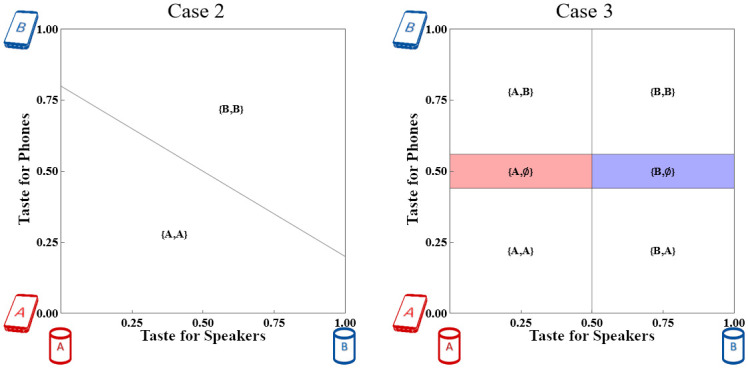
Consumer market shares of Weak taste devices with *T*_*S*_ = ^3^/_16_ and *T*_*P*_ = ^5^/_16_ (violating Assumption 2 as in Proposition 3). The horizontal axis measures taste for Speakers while the vertical axis measures taste for Phones. Shaded areas represent sales of a new platform device as a substitute for the Phone.

Proposition 3 implies that the new platform device ceases to be a complement to the Phone, and it starts disrupting the Phone market. The intuition behind this result is that the increased misfit cost creates a group of Consumers with intermediate *y* indices would prefer an outside option to the Phones offered. This outside option would be the modern version of a feature phone (including some social media features, but no app ecosystem). Fig 6 shows that depending on model parameters, some of these Consumers would maximize their utility with {A,∅} and {B,∅}, which never had positive market share in any other Case analyzed earlier.

Note that violating assumption (A2) means that the Weak taste device market could tip into Narrow adoption, which would lead to a positive market share for {∅,∅} (a Consumer with a feature phone and no smart home appliance) which also never had positive market share in any previous Case.

### 4.6 Comparison of competition case outcomes

We summarize firm prices and profits across all cases in Table 3. The equilibrium prices remain stable for every case, *T*_*P*_ for Phones and *T*_*W*_ for Watches, because cross-side network effects are competed away as shown above. Meanwhile, the revenue and, therefore, the profit varies depending on how many Consumers purchase at the equilibrium price.

**Table 3:** Summary of market outcomes.

Case	Profit from Phone Consumers	Profit from Watch Consumers	Profit from Phone Developers	Profit from Watch Developers
Case 1, Baseline	$\frac{T_{P}}{2}$	0	vP2+nP42tP	0
Case 2, Narrow Adoption	$\frac{T_{P}}{2}$	$\frac{\left({40T}_{P}-20T_{W}+9\right)\left(9-20T_{W}\right)}{1600T_{P}}$	vP2+nP42tP	vW2+nW42tW
Case 2, Wide Adoption	$\frac{T_{P}}{2}$	$\frac{{40T}_{P}\left(9-20T_{W}\right)-\left(9-40T_{W}\right)\left(27-80T_{W}\right)}{1600T_{P}}$	vP2+nP42tP	vW2+nW42tW
Case 3, Narrow Adoption	$\frac{T_{P}}{2}$	$\frac{\left({9-20T}_{W}\right)}{20}$	vP2+nP42tP	vW2+nW42tW
Case 3, Wide Adoption	$\frac{T_{P}}{2}$	$\frac{T_{W}}{2}$	vP2+nP42tP	vW2+nW42tW

Adoption will always be Wide for the weak-taste device, and in principle could be Narrow or Wide for a strong-taste device. If Firms are free to choose the strength of tastes (*i*.*e*., misfit costs) for the baseline Phone, then it is profit-maximizing to set it at the maximum strength possible while maintaining full Consumer market coverage. In such a case, a strong-taste device would always have Narrow adoption.

Note that the expressions in the Profit from Watch Consumers are all equal to or less than *T*_*W*_/2. This upper limit is reached under Wide adoption with very low misfit costs in Case 2, and under any Wide adoption in Case 3.

**Fig 7:**
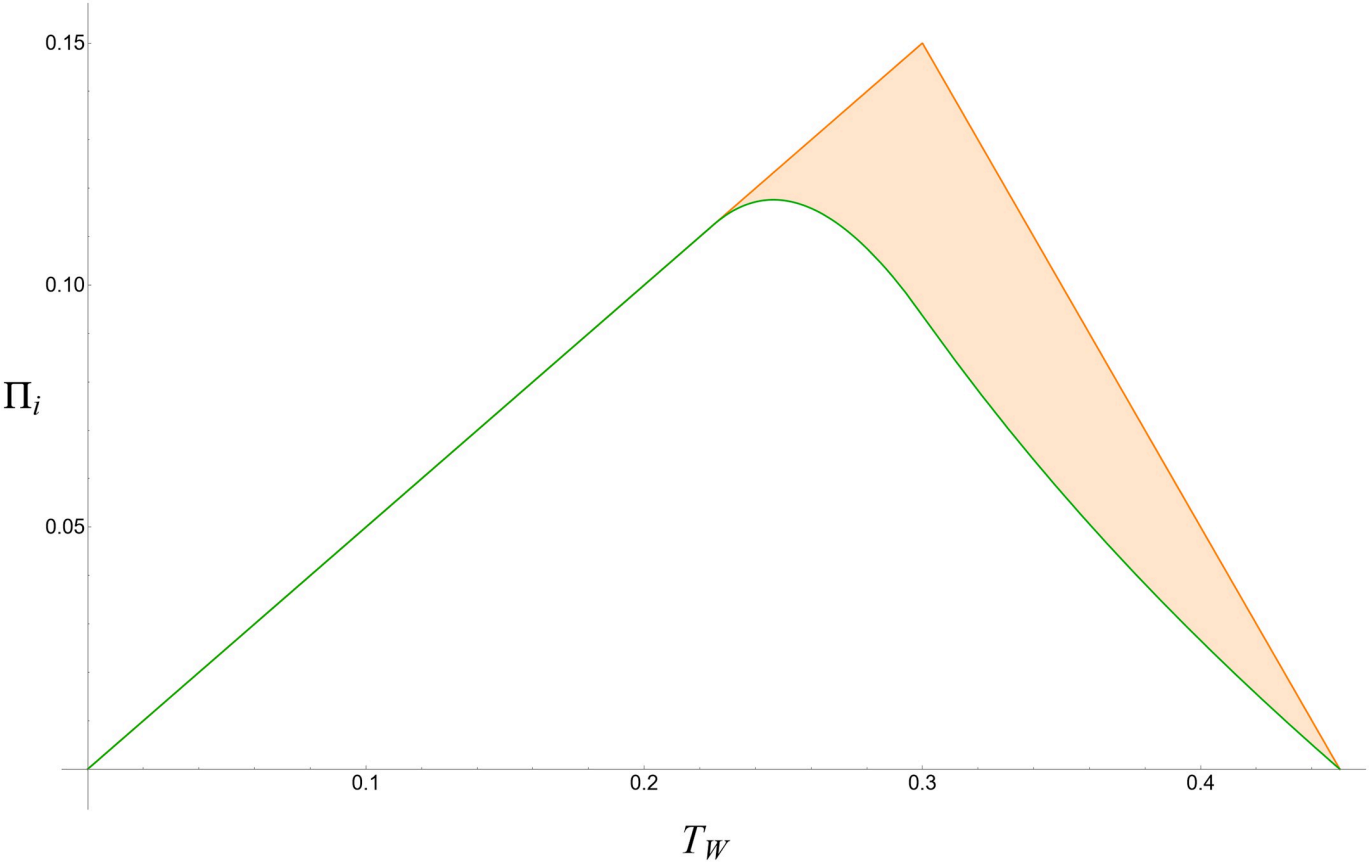
Firm profit from Watches in Case 2 (green, unshaded) and Case 3 (orange, shaded) across different values of *T*_*W*_. To stay consistent with Definition 1, *T*_*P*_ is treated as ^9^/_10_ of *T*_*W*_.

We illustrate the Firm’s profit from Watches in Case 2 and Case 3 in Fig 7. Maximum profit under Case 2 is realized at TW=9-4TP32, and the maximum profit under Case 3 is realized at *T*_*W*_ = ^3^/_10_ (the threshold between Wide and Narrow adoption). Case 3’s incremental profit (the shaded region) arises from selling Watches to users of the rival Phone.

The value of the exogenous parameter *T*_*W*_ determines the Firm’s optimal choice between going to market with Case 2 (Watches that work only with Phones from the same Firm) or Case 3 (standalone Watches). To the extent that the Firm can influence *T*_*W*_ through research and development efforts, Proposition 4 serves as a guide.

**Proposition 4 (Choice between Case 2 and Case 3):** Case 3 profit is always higher than Case 2 profit under Narrow adoption as defined in Definition 3. The profit for each Case is equal under Wide adoption when TW≤910-2TP-2431600TP; otherwise Case 3 results in higher profit.■

While Case 3 weakly dominates Case 2 *ex ante* for strong-taste devices, if the Firm expects *T*_*W*_ to be low and *T*_*P*_ not to be very low, then Case 2 may prove to be the less expensive technology to develop without losing any potential profit. On the other hand, if Narrow adoption is expected, then Case 3 is profit maximizing.

For weak-taste devices, the Consumer market is always fully covered. This ensures the low *T*_*W*_ condition from the previous paragraph, and there is no profit difference between Case 2 and Case 3.

## 5. Model extension

What is the effect of designing the Watch to mimic the look-and-feel user experience of the Phone? Some models of Speakers already include home-screen-style displays, and Firms could add a collection of Phone-like gestures to Watches. We explore these converging devices in an extension of Case 3, which we will call Case 3A. Convergence introduces a correlation into the misfit costs for both devices from the same Firm. For a given fraction of Phone-ness 0 ≤ *α* < ^1^/_2_, the Consumer utility functions for these watches are:

$$\begin{array}{l} u_{WA}\left(x,y\right)=u_{W}+N_{W}∙q_{WA}-\left[\sqrt{1-\alpha^{2}}∙x∙T_{W}+\alpha ∙y∙T_{P}\right]\\ u_{WB}\left(x,y\right)=u_{W}+N_{W}∙q_{WB}-\left[\sqrt{1-\alpha^{2}}∙\left(1-x\right)∙T_{W}+\alpha ∙\left(1-y\right)∙T_{P}\right] \end{array}$$

Setting *α* = 0 recreates Case 3, and we find that a strictly positive *α* retains the qualitative features of our model. It does, however, induce changes at the margins that Firms should consider when designing the Watch.

The presence of *α* slightly affects the feasible range of network effects, so we restate assumption (A4) taking into account nonzero *α* and this is assumption (A4A): Consumer network effects for Watches *N*_*W*_ are between the lower limit 2tW∙α∙TP+2TW-2uWnW+2vW and the upper limit 2tW∙α∙TP+2∙1+1-α2∙TW-2uWnW+2vW.■

Definition 1 continues to hold in Case 3A, except that now the indifference line is no longer vertical. One must measure *x* at the point where the indifference line crosses *y* = ^1^/_2_. The threshold taking *α* into account is NW<2tW∙α∙TP+2+1-α2∙TW-2uWnW+2vW.

We summarize the impact of incorporating the Phone’s look-and-feel into the watch in Proposition 5.

**Proposition 5 (Market outcome in Case 3A):** Under Wide adoption of Watches per Definition 3, increasing misfit-relevant Phone features in the Watch increases adoption of single-vendor systems at the expense of mixed-vendor systems with overall sales remaining constant. Under Narrow adoption of Watches, increasing misfit-relevant Phone features decreases sales of Watches.■

The intuition behind this result is that increasing *α* tilts all non-horizontal indifference lines counter-clockwise, with {A,A}~{∅,A} anchored at a point near *y* = 0 and {B,B}~{∅,B} anchored at a point near *y* = 1. If Watch adoption is still Wide measured at *y* = ^1^/_2_ then {A,A}~{∅,A} is irrelevant and the {A,A}~{B,A} indifference line passes directly through the point (^1^/_2_, ^1^/_2_). Rotating such an indifference line counter-clockwise will sweep previous {B,A} Consumers into {A,A} as well as {A,B} Consumers into {B,B}. Meanwhile {A,A}~{A,B} and {B,B}~{B,A} remain fixed at *y* = ^1^/_2_ so that mixed-vendor systems receive no new adopters.

**Fig 8:**
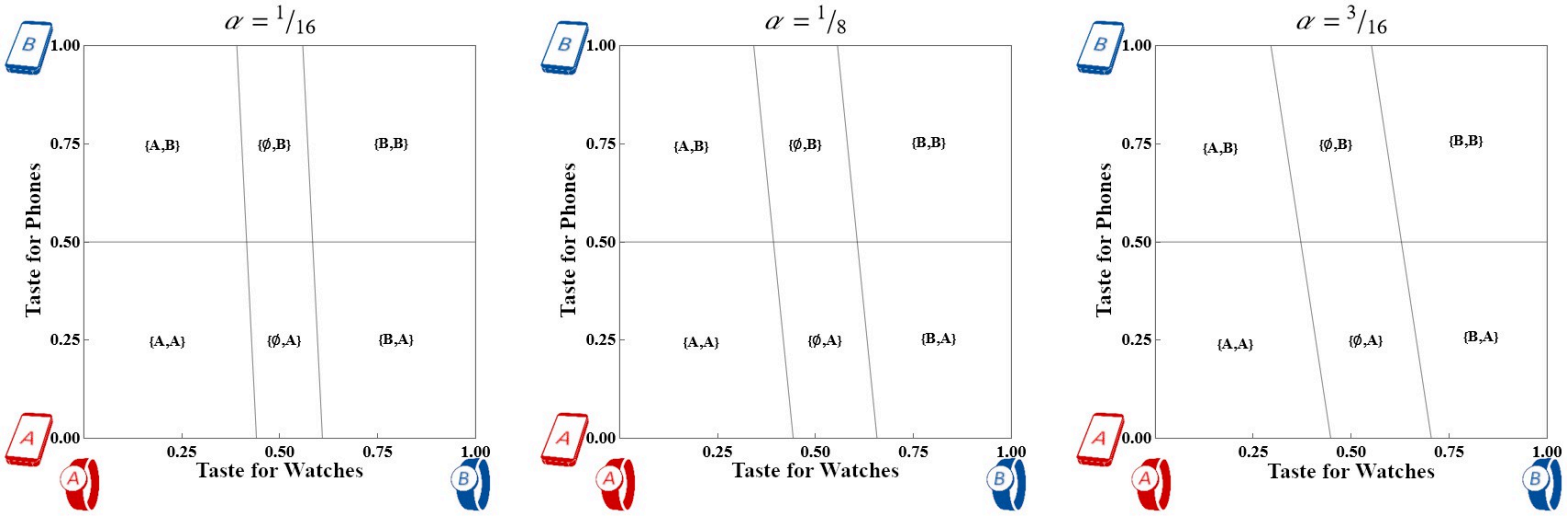
Consumer market shares for Strong devices with *T*_*W*_ = ^5^/_16_ and *T*_*P*_ = ^1^/_4_ when the Watch device is designed with three different values of *α*. The horizontal axis measures taste for pure Watches while the vertical axis measures taste for Phones.

On the other hand, if Watch adoption is Narrow when measured at *y* = ^1^/_2_ then rotating the indifference lines widens the region where Consumers forego purchasing Watches, as shown in Fig 8. Formally, the market share of the new device, 9-20TW-10TPα201-α2, is decreasing in *α*.

Overall, the impact of Phone-like features depends on the extent of adoption of the device. Under Wide adoption, increasing *α* homogenizes the market by nudging Consumers toward pairing devices from the same Firm. On the other hand, under Narrow adoption, increasing *α* makes the Phone-like Watch unpalatable to more and more Consumers and decreases sales of the device. Note that *α* figures into the level of adoption at *y* = ^1^/_2_, so it is possible for a previously Wide-adopting market to tilt into a Narrow-adopting one.

We expect that wearables will follow a path toward high differentiation while smart home appliances will follow a path toward modest differentiation, but it is conceivable that either or both predictions may prove inaccurate. The key driver for market outcomes in Case 3A is whether differentiation precludes full coverage of the Consumer market.

## 6. Discussion & conclusions

We discuss theoretical and managerial implications and identify opportunities for future research.

### 6.1 Research findings and theoretical implications

We propose a novel analytical model that features two firms. In the baseline case (Case 1), the firms sell only smartphones, a platform device connecting consumers and developers of apps. They exhibit cross-side network effects from developers to consumers and from consumers to developers, but otherwise, they behave in a manner that is not affected by other networked markets. Complementary hardware such as earpods and pedometers did exist. However, these goods did not exhibit the cross-side network effects that would significantly affect the adoption of one smartphone over another.

In the subsequent competition cases, firms introduce a complementary platform device (such as a smartwatch), so each firm becomes a *multiplatform firm* orchestrating a *multiplatform ecosystem*. The transition from a platform firm to a multiplatform firm affects the adoption patterns of all platform devices and firm profits. The precise effect depends on the class of the new platform device, and firms’ choices about the design of the new platform devices, which determines the competition case.

In the second competition case (Case 2), firms introduce a new platform device that might provide minimal functionality on its own, but functions fully when paired with a smartphone from the same vendor. For example, an early generation Apple Watch was not capable of much without a constant connection to an iPhone.

The extent of adoption of the new platform device among consumers depends on the relative strength of network effects and tastes, with items that exhibit low misfit costs due to weak tastes (*i*.*e*., smart home appliances) enjoying wide or even universal adoption. Items that exhibit high misfit costs due to strong tastes (*i*.*e*., wearables) could end up with any level of adoption from niche to wide, depending on the model parameters.

In the third case, the newer device has reasonable standalone functionality. An example of a Case 3 device is a smartwatch that does not require pairing with a smartphone. Unlike Case 2, a consumer who prefers devices from different vendors can expect each to work properly. Items that won wide adoption in Case 2 have offerings spaced closely enough that every consumer has at least one combination of devices that yields positive utility at equilibrium prices.

We also explore an extension in which consumers’ preferences for the two devices from the same firm are positively correlated. The extension complicates the model’s mathematical statements considerably, but the results are qualitatively similar to the results without correlation. The indifference lines shift to benefit single-vendor systems, however, the intuition behind Narrow or Wide adoption driving final market outcomes remains.

When could a new platform device start disrupting the incumbent smartphone market? We show that this depends on the competition case and the new platform device class, which is determined by the relative strength of consumer tastes. We show that the threat from Strong taste devices (like wearables) is probably hypothetical. However, the threat from Weak taste devices (like smart home appliances) is plausible given the historical trends in industries where technical capability grows faster than consumers’ collective ability to harness that capability [37]. The new platform device becomes good enough for most consumers, and the incumbent device withers to perhaps just a remote peripheral of the smart home appliance. If smart appliances become common in homes, vehicles, and places of business, then the smartphone, today’s essential must-have device, may become suddenly redundant.

### 6.2 Managerial implications

A *multiplatform firm* offers a *system of platforms* and orchestrates a *multiplatform ecosystem*. These concepts constitute a significant conceptual contribution to the managerial understanding of platform strategies and ecosystems [38–43].

Our model and analysis demonstrate how the strategic imperatives of multiplatform firms differ from those of platform firms. A platform firm coordinates a platform ecosystem that consists of users, application developers and other providers of complementary products and services that affect the value of its platform. The firm seeks to enlarge the value of the platform ecosystem and maximize the platform profit. It needs to consider the interaction between the sides of the platform (cross-side network effects), and competition from other platform firms.

In contrast, a *multiplatform firm* offers two (or more) platforms, and it coordinates a multiplatform ecosystem. The firm seeks to enlarge the value of the whole multiplatform ecosystem, while it maximizes its profit from all the platforms. The firm needs to consider the interaction between the sides of the platform (cross-side network effects), the interaction between its platform devices, and competition from other platform or multiplatform firms.

Managers of multiplatform firms must be aware of several important issues, starting with the preferences of consumers and developers for each platform device and the strength of cross-side network effects. When a firm transitions from a platform firm to a multiplatform firm, consumer adoption patterns and competition change. It is essential to know the class of the new platform device the firm offers: we identified two classes of devices differentiated by consumer tastes that are Strong or Weak relative to smartphones, empirically observable as Narrow or Wide adoption, respectively.

Managers should also be aware of distinct competition Cases that capture the firm strategies in the consumer IoT landscape. Our analysis showed that competition cases matter because strategic behavior and outcomes differ across cases. In Case 2, the functioning of the new platform device depends on the matching smartphone. In Case 3 the new platform device is standalone, which also opens the possibility that a consumer may not purchase the smartphone, or a consumer may mix-and-match devices across vendors. Firms may also design their new platform device to be more phone-like (Case 3A).

The current generation of smartwatches drives sales primarily through complementarity with smartphones (Case 2), but not all consumers find this combination compelling. For example, one may prefer the iPhone to an Android phone, yet simultaneously prefer an Android Wear watch to the Apple Watch. Tying smartwatches to smartphones in this way, therefore, generates some deadweight loss. Standalone smartwatches hold the promise of serving many of these consumers.

It is important to emphasize that competition cases reflect strategic technology choices that the firms make. Nothing inherent in IoT technology demands that devices evolve from Case 1 to Case 2 to Case 3 to Case 3A. An IoT firm may foresee strong tastes for both platforms and proceed directly from single-platform Case 1 to multiplatform Case 3. Alternatively, a firm that foresees no additional profit moving from Case 2 to Case 3 will not do so unless compelled to do so by regulators.

Firms must keep the strength of consumer tastes in mind when moving look-and-feel features from one device to another. When consumer misfit costs (and therefore, platform adoption decisions) center on the smartphone, firms are no worse off for incorporating smartphone features to provide a consistent experience across devices. On the other hand, when misfit costs are higher for the complement (*e*.*g*., wearables), consumers may rebel against losing the complement’s distinctiveness.

A firm must be prepared for the possibility that a new platform device may start disrupting the incumbent platform. That is especially true when the firm falls into the trap of overinvesting in increasing the misfit/transportation cost for the incumbent platform device. Managing that transition will require maintaining control of all the platforms in their *system of platforms*, foreclosing the option of spinning off non-core businesses.

Overall, managers in the technology industry will benefit by applying *multiplatform thinking* to strategic issues they face.

### 6.3 Future research opportunities

Future research could extend the proposed analytical framework in several directions. More research is needed on the economics and strategic implications of multiplatform firms and multiplatform ecosystems. Future research could examine inter-temporal competition, competition between multiple generations of the same device, and more nuanced strategies for attracting developers, such as exclusive contracting.

IoT provides a complex and fast-changing landscape for vendors of digital platforms, developers of complementary apps and services, and consumers. Future economics of IT work could consider challenges such as security and privacy [44] and other strategic IoT effects.

### 6.4 Concluding remarks

Our research introduces and formalizes the concept of a *multiplatform firm* that offers a *system of platforms* and orchestrates a *multiplatform ecosystem*. We propose a novel analytical model of competing multiplatform firms inspired by the consumer IoT landscape. The article contributes to the economics of platforms and provides lessons for managers of multiplatform firms. Moreover, our work contributes to the consumer IoT economics literature and sheds light on consumer IoT firm strategies and competition outcomes.

## Supporting information

S1 Appendix Analytical proofs. (DOCX)
