# Supplementary material for: Competition of multiplatform firms: Implications for the Internet of Things
Source: PLoS One. 2024 May 14;19(5):e0300522. doi: 10.1371/journal.pone.0300522 (PMC11093345; doi:10.1371/journal.pone.0300522)
Supplement: S1 Appendix — (DOCX) [file pone.0300522.s001.docx]

**Appendix: Analytical proofs**

**Proof of Lemma 1:** Full market coverage of Consumers occurs if utility at the equilibrium price is positive at *y* = ^1^/_2_. Determining utility requires first determining cross-side network effects, which requires knowing the fraction of Developers on the platform.

With costless multihoming, each Firm prices as a monopolist for Developers. Substituting one-half for Consumer market share into the Developer utility function, we arrive at the market share assuming an interior solution.

$y^{*}=\frac{v_{P}+\frac{n_{P}}{2}-p_{PA}}{t_{P}}$

The Firm then sets a profit-maximizing price.

$\max_{p_{PA}} p_{PA}\cdot\frac{v_{P}+\frac{n_{P}}{2}-p_{PA}}{t_{P}}$

The first-order condition provides the profit-maximizing price and market share.

$\begin{matrix} \frac{v_{P}+\frac{n_{P}}{2}-{2p}_{PA}}{t_{P}}=0 \\ p_{PA}^{*}=\frac{v_{P}}{2}+\frac{n_{P}}{4} \\ y^{*}=\frac{\frac{v_{P}}{2}+\frac{n_{P}}{4}}{t_{P}} \end{matrix}$

Substituting in the known market shares, the full market condition becomes

$\begin{matrix} u_{P}+N_{P}\cdot\left( \frac{\frac{v_{P}}{2}+\frac{n_{P}}{4}}{t_{P}} \right)-\frac{1}{2}T_{P}>0 \\ T_{P}<2u_{P}+\frac{N_{P}}{t_{P}}\cdot\left( v_{P}+\frac{n_{P}}{2} \right) \end{matrix}$

which is the condition to be proven.■

**Proof of Proposition 1:** Given the optimal price to Consumers *T_W_* it follows that the indifference line {A,A} ~ {∅,A} will be vertical at $x=\frac{\left( 9-20T_{W} \right)}{20T_{W}}$. Under Narrow adoption per Definition 3, that is *x* ≤ ^1^/_2_ or equivalently *T_W_* ≥ ^3^/_10_, the indifference line {A,A} ~ {∅,B} lies at $y=\frac{\left( 20T_{P}-20T_{W}+9 \right)}{40T_{P}}-\left( \frac{T_{W}}{2T_{P}} \right)x$ tracing out a trapezoid with area $\frac{\left[ \left( 40T_{P}-20T_{W}+9 \right)\left( 20T_{W}-9 \right) \right]}{1600T_{P}T_{W}}$. Under Wide adoption, the indifference lines {A,A} ~ {∅,A}, {A,A} ~ {B,B}, and {A,A} ~ {∅,B} trace out a polygon with area $\frac{\left( 40\left[ T_{P}\left( 9-20T_{W} \right)+T_{W}\left( 153-260T_{W} \right) \right]-891 \right)}{1600T_{P}T_{W}}$. Under the conditions of Definition 1, namely *T_W_* > *T_P_* > 0, both market shares are deterministically ^1^/_2_ or less. The optimal price and market share for Developers are derived in Lemma 1.

To determine whether any Consumers purchase a Phone but no Watch, we consider system {∅,A}, which has simpler notation than {∅,B}. Consider the Consumer indifferent between systems {A,A} and {∅,A}:

$$\begin{matrix} U_{,A}\left( x,y \right)=U_{A,A}\left( x,y \right) \\ u_{P}+N_{P}q_{PA}-yT_{P}-T_{P}=u_{W}+N_{W}q_{WA}-xT_{W}+u_{P}+N_{P}q_{PA}-yT_{P}-\left( T_{P}+T_{W} \right) \\ 0=u_{W}+N_{W}q_{WA}-xT_{W}-T_{W} \end{matrix}$$

and between systems {B,B} and {∅,A}:

$$\begin{matrix} U_{,A}\left( x,y \right)=U_{B,B}\left( x,y \right) \\ u_{P}+N_{P}q_{PA}-yT_{P}-T_{P}=u_{W}+N_{W}q_{WB}-\left( 1-x \right)T_{W}+u_{P}+N_{P}q_{PB}-\left( 1-y \right)T_{P}-\left( T_{P}+T_{W} \right) \\ 0=u_{W}+N_{W}q_{WA}-\left( 2-x \right)T_{W}-{\left( 1-2y \right)T}_{P} \end{matrix}$$

Substituting in the known Developer market shares yields solutions for *x* and *y*:

$$\begin{matrix} x=\frac{u_{W}+N_{W}\left( \frac{\frac{v_{W}}{2}+\frac{n_{W}}{4}}{t_{w}} \right)}{T_{W}}-1 \\ y=\frac{1}{2}-\frac{u_{W}+N_{W}\left( \frac{\frac{v_{W}}{2}+\frac{n_{W}}{4}}{t_{w}} \right)-2T_{W}}{2T_{P}}-\frac{T_{W}}{2T_{P}}x \end{matrix}$$

A sufficient condition for system {∅,A} to have positive sales is *x* < 1 and *y* > 0. The *x* condition is straightforward:

$$\begin{matrix} \frac{u_{W}+N_{W}\left( \frac{\frac{v_{W}}{2}+\frac{n_{W}}{4}}{t_{w}} \right)}{T_{W}}-1<1 \\ N_{W}<\frac{4t_{W}\left( 2T_{W}-u_{W} \right)}{n_{W}+2v_{W}} \end{matrix}$$

Substituting the definition of *x* into the definition of *y* yields a solution for *y* that is always positive with positive *T_P_* and *T_W_*, so it adds no additional constraint to the condition already derived.■

**Proof of Lemma 2:** Without loss of generality, we consider system {B,A}. This system will have positive sales if the indifference line {B,A}~{B,B} crosses the {A,A}~{B,A} line (or the {∅,A}~{B,A} line under Narrow adoption) at a point with *x* < 1 and *y* > 0.

First, under Wide adoption, most terms in the {A,A}~{B,A} line cancel out, leaving a deterministic solution at *x* = ^1^/_2_. The *N_W_* condition for Wide adoption is tighter than the condition under consideration for this Lemma, so we shall not consider this case further.

Second, under Narrow adoption the {∅,A}~{B,A} line is:

$$\begin{matrix} u_{P}+N_{P}q_{PA}-yT_{P}-T_{P}=u_{P}+N_{P}q_{PA}-yT_{P}+u_{W}+N_{W}q_{WB}-\left( 1-x \right)T_{W}-\left( T_{P}+T_{W} \right) \\ 0=u_{W}+N_{W}\left( \frac{\frac{v_{W}}{2}+\frac{n_{W}}{4}}{t_{W}} \right)-\left( 1-x \right)T_{W}-T_{W} \\ x=2-\frac{u_{W}+N_{W}\left( \frac{\frac{v_{W}}{2}+\frac{n_{W}}{4}}{t_{W}} \right)}{T_{W}} \end{matrix}$$

which leads to the condition

$$\begin{matrix} 2-\frac{u_{W}+N_{W}\left( \frac{\frac{v_{W}}{2}+\frac{n_{W}}{4}}{t_{W}} \right)}{T_{W}}<1 \\ N_{W}>\frac{4t_{W}\left( T_{W}-u_{W} \right)}{n_{W}+2v_{W}} \end{matrix}$$

Third, most terms in the {B,B}~{B,A} line cancel out, leaving a deterministic solution at *y* = ^1^/_2_.

Finally, since the *y* condition is always met and $N_{W}>\frac{4t_{W}\left( T_{W}-u_{W} \right)}{n_{W}+2v_{W}}$ guarantees the *x* condition is met, the Lemma is proved.■

**Proof of Proposition 2:** Under Wide adoption, the indifference lines {A,A}~{B,A} and {B,B}~{A,B} always occur at *x* = ^1^/_2_, while the indifference lines {A,A}~{A,B} and {B,B}~{B,A} always occur at *y* = ^1^/_2_. Such an arrangement results in full coverage of the Consumer market with equal market share for each system.

Under Narrow adoption, the indifference lines {A,A}~{A,B} and {B,B}~{B,A} still always occur at *y* = ^1^/_2_. What remains to be proven is that the vertical, collinear lines {A,A}~{∅,A} and {A,B}~{∅,B} are the same distance from *x* = 0 that the vertical, collinear lines {B,B}~{∅,B} and {B,A}~{∅,A} are from *x* = 1.

The first indifference line lies where the marginal consumer receives zero utility from adding a watch to her system.

$$\begin{matrix} u_{W}+N_{W}\left( \frac{\frac{v_{W}}{2}+\frac{n_{W}}{4}}{t_{W}} \right)-xT_{W}-T_{W}=0 \\ x=\frac{u_{W}+N_{W}\left( \frac{\frac{v_{W}}{2}+\frac{n_{W}}{4}}{t_{W}} \right)}{T_{W}}-1 \end{matrix}$$

Under the condition from Definition 1, we know that this *x* < ^1^/_2_. We solve the second indifference line for “1 – x” to show that its width is identical.

$$\begin{matrix} u_{W}+N_{W}\left( \frac{\frac{v_{W}}{2}+\frac{n_{W}}{4}}{t_{W}} \right)-\left( 1-x \right)T_{W}-T_{W}=0 \\ 1-x=\frac{u_{W}+N_{W}\left( \frac{\frac{v_{W}}{2}+\frac{n_{W}}{4}}{t_{W}} \right)}{T_{W}}-1 \end{matrix}$$

The adoption regions for all four systems have the same height and same width, and therefore the same area.■

**Proof of Proposition 3:** Violating Assumption 2 leads to Narrow adoption of Phones, opening a gap between {A,A} and {A,B} as well as between {B,B} and {B,A}. The indifference lines {A,A}~{A,∅} and {∅,A}~{∅,∅} occur at some 0 < *y* < ^1^/_2_ while {B,B}~{B,∅} and {∅,B}~{∅,∅} occur at some ^1^/_2_ < *y* < 1.

By Definition 2, the new platform device has a lower misfit cost, and therefore the indifference line {A,∅}~{∅,∅} occurs at some *x* > 0, and happens to be further away from the *x* = 0 line than the {A,A}~{A,∅} line was from the *y* = 0 line. By similar reasoning, the indifference line {B,∅}~{∅,∅} occurs at some *x* < 1. If the new device’s misfit costs remain Wide, then the binding indifference line is {A,∅}~{B,∅}, which occurs at exactly *x* = ^1^/_2_.

The above conditions create regions with a nonzero area for systems {A,∅} and {B,∅}.■

**Proof of Proposition 4:** Under Narrow adoption as defined in Definition 3, the market share for Case 3 is the rectangle bound by *x* = 0, *y* = 0, *y* = 1, and {A,A}~{∅,A} which has an area of (9 ‑ 20*T_W_*)/20*T_W_*. This area is greater than the market share under Case 2, a quadrilateral bound by *x* = 0, *y* = 0, {A,A}~{∅,B}, and {A,A}~{∅,A} which is a subset of the above rectangle and has an area of $\frac{\left( 40T_{P}-20T_{W}+9 \right)\left( 9-20T_{W} \right)}{1600T_{P}T_{W}}$.

Under Wide adoption with symmetric Firms, the maximum market share is ^1^/_2_ with a corresponding revenue from Watches of *T_W_*/2. Case 3 will always reach these maxima under Wide adoption. The market share for Case 2 under Wide adoption is the pentagon bound by *x* = 0, *y* = 0, {A,A}~{∅,B}, {A,A}~{B,B}, and {A,A}~{∅,A} if the intersection of {A,A}~{∅,B} and {A,A}~{B,B} is within the Hotelling square. This region has a total area of $\frac{\left[ 40T_{P}\left( 9-20T_{W} \right)-\left( 9-40T_{W} \right)\left( 27-T_{W} \right) \right]}{1600T_{P}T_{W}}$ which is deterministically less than ^1^/_2_. If the intersection of {A,A}~{∅,B} and {A,A}~{B,B} falls outside the Hotelling square, then the market share is exactly ^1^/_2_.

The condition under which the intersection occurs within the Hotelling square simplifies to $T_{W}>\frac{\left( \frac{9}{10}-2T_{P}-\frac{243}{1600T_{P}} \right)}{1600T_{P}T_{W}}$, therefore Case 2 and Case 3 yield the same market share under the condition $T_{W}\leq\left( \frac{9}{10}-2T_{P}-\frac{243}{1600T_{P}} \right)$ stated in the Proposition. ■

**Proof of Proposition 5:** There are three scenarios to consider when describing the impact of *α*.

The first scenario is the most straightforward. If *α* increases enough to tip Wide adoption into Narrow adoption, sales of watches have decreased by definition, and the Proposition holds.

The second scenario is that the {∅,A} and {∅,B} regions exist due to Narrow adoption. We must show that these regions widen as *α* increases. Although the slopes of indifference lines {A,A}~{∅,A} and {B,B}~{∅,B} change with *α*, the lines remain parallel so it is sufficient to measure the *x* distance between them at any given point (it would be multiplied by its constant height of one to find the area of Phone-only systems). This distance is

$$\Delta x=\frac{2t_{W}\left[ \alpha T_{P}+\left( 2+\sqrt{1-\alpha^{2}} \right)T_{W}-2u_{W} \right]-N_{W}\left( n_{W}+2v_{W} \right)}{2T_{W}t_{W}\sqrt{1-\alpha^{2}}}$$

The derivative of this distance with respect to *α* is not deterministically positive at every point (it turns negative as *α* nears ^1^/_2_ if *T_W_* is drastically higher than *T_P_*), but the distance is always minimized by setting *α* = 0.

$$\underset{\alpha}{arg max} \frac{2t_{W}\left[ \alpha T_{P}+\left( 2+\sqrt{1-\alpha^{2}} \right)T_{W}-2u_{W} \right]-N_{W}\left( n_{W}+2v_{W} \right)}{2T_{W}t_{W}\sqrt{1-\alpha^{2}}}=0$$

The third scenario is that Wide adoption causes the single-vendor systems and mixed-vendor systems to divide the entire Consumer market. The colinear indifference lines {A,A}~{B,A} and {B,B}~{A,B} pass through the point (^1^/_2_, ^1^/_2_), so we need only show that the line sweeps to the right at *y* = 0 (which implies that it sweeps to the left at *y* = 1). Substituting *y* = 0 into {A,A}~{B,A} yields:

$$x=\frac{u_{W}+N_{W}\frac{u_{W}+N_{W}\left( \frac{\frac{v_{W}}{2}+\frac{n_{W}}{4}}{t_{W}} \right)-T_{W}}{T_{W}}}{T_{W}\sqrt{1-\alpha^{2}}}$$

Increasing *α* clearly decreases the denominator, which increases *x* at *y* = 0.

Having proven all three possible scenarios, the Proposition is proven.■
